# Laparoscopic resection of a gastrointestinal stromal tumor of the lower rectum in a patient with coronary artery disease following long-term neoadjuvant imatinib treatment and anticoagulation therapy

**DOI:** 10.1186/1477-7819-12-211

**Published:** 2014-07-15

**Authors:** Hiroaki Nozawa, Takamitsu Kanazawa, Toshiaki Tanaka, Masao Takahashi, Soichiro Ishihara, Eiji Sunami, Joji Kitayama, Masako Ikemura, Issei Komuro, Toshiaki Watanabe

**Affiliations:** 1 Department of Surgical Oncology, The University of Tokyo, 7-3-1 Hongo, Bunkyo-ku, Tokyo 113-8655, Japan; 2 Department of Cardiovascular Medicine, The University of Tokyo, 7-3-1 Hongo, Bunkyo-ku, Tokyo 113-8655, Japan; 3 Department of Pathology, The University of Tokyo, 7-3-1 Hongo, Bunkyo-ku, Tokyo 113-8655, Japan

**Keywords:** Anti-platelet therapy, Gastrointestinal stromal tumor, Imatinib, Laparoscopic surgery, Percutaneous coronary intervention

## Abstract

Surgery is the mainstay of treatment for gastrointestinal stromal tumors (GISTs). However, complete resection of rectal GISTs is sometimes difficult because of bulkiness and/or anatomical reasons. Neoadjuvant imatinib therapy has gained attention as an alternative treatment to increase the chance of *en bloc* resection of rectal GISTs, although it usually takes several months. In this case report, we first demonstrated that neoadjuvant imatinib therapy can be performed safely not only to downsize tumors, but also to allow adequate time for the effective treatment of major comorbid illnesses. A 74-year-old man was diagnosed with a 45 mm GIST of the lower rectum. He also had severe stenosis in the proximal segment of the left anterior descending coronary artery. Following the implantation of a drug-eluting stent, the patient received imatinib together with dual anti-platelet therapy for 12 months without obvious side effects. Follow-up image studies revealed tumor shrinkage as well as stent patency. *En bloc* resection of the GIST was performed laparoscopically, which preserved the anus. The patient is currently alive without any evidence of relapse for 12 months after surgery.

## Background

Gastrointestinal stromal tumors (GISTs) of the lower rectum often require invasive surgery such as abdominoperineal excision and multivisceral resection [1]. The preoperative administration of imatinib, a selective tyrosine kinase inhibitor, for rectal GISTs with the aim of anus-preserving surgery is still a challenging therapy that typically takes several months [2]. We herein described a patient with a GIST initially involving the lower rectum and anal canal who received imatinib treatment for one year and concomitant anti-coagulant therapy after the implantation of a drug-eluting stent (DES) for coronary artery disease. Marked tumor shrinkage allowed low anterior resection to be performed laparoscopically while preserving the anus.

## Case Presentation

**Figure 1:**
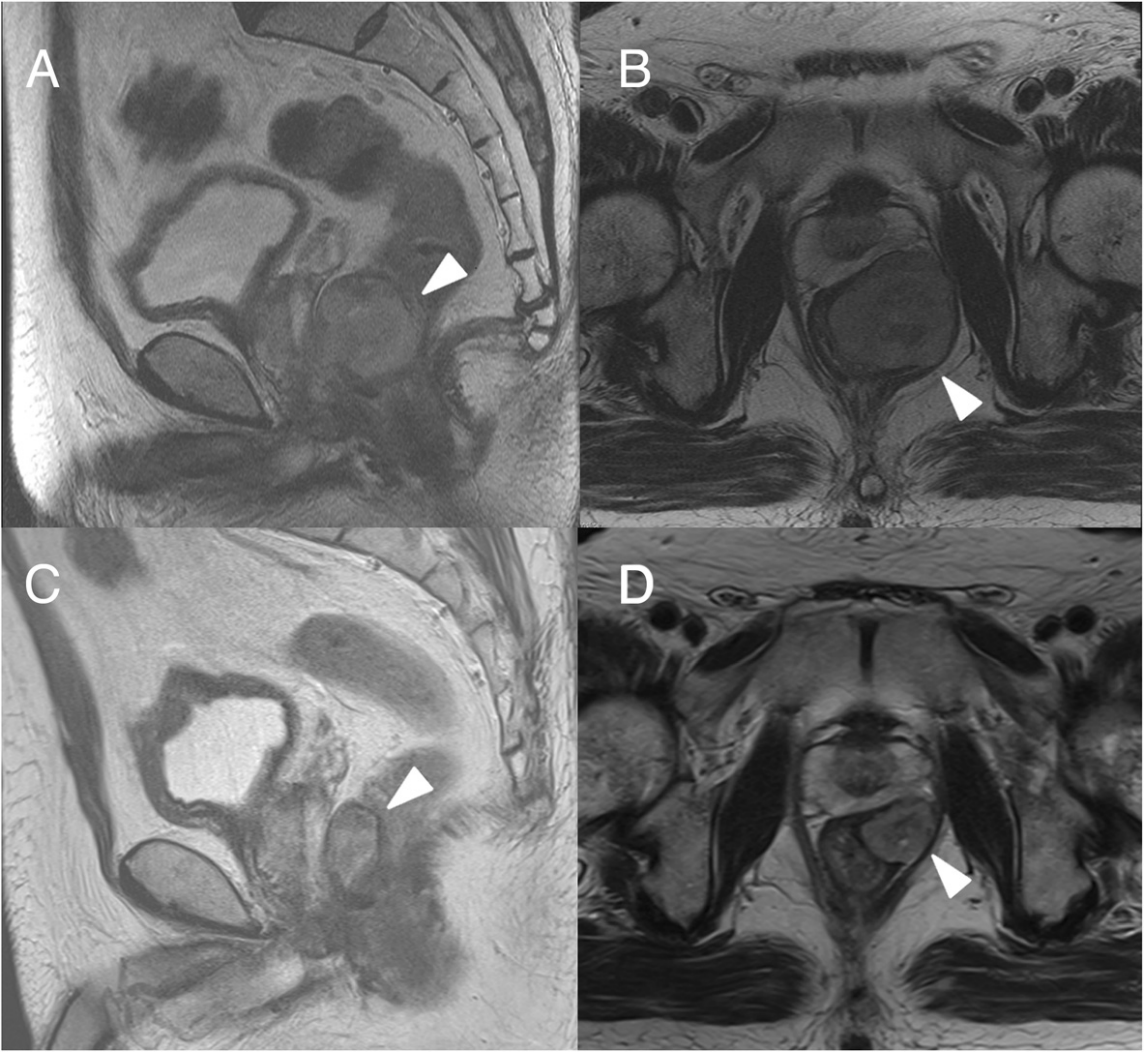
**MRI examination. (A, B)** Pelvic MRI before imatinib therapy A 45 mm tumor was detected in the lower rectum adjacent to the posterior wall of the prostate (arrowhead). **(C, D)** Pelvic MRI after imatinib therapy for one year. The tumor shrank to 24 mm in diameter (arrowhead). (**A**, **C**: Coronary view, **B**, **D**: Sagittal view).

**Figure 2:**
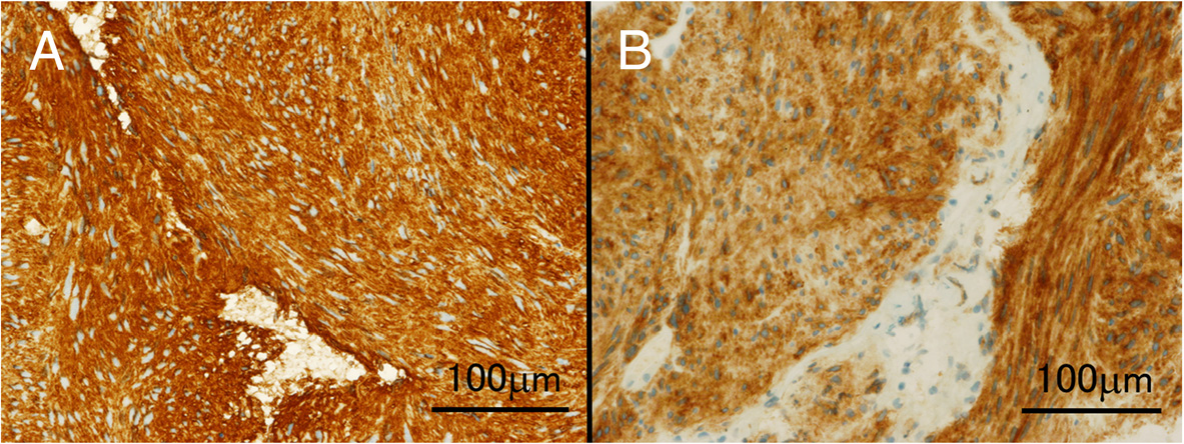
**Histological examination of the biopsied specimen. (A)** CD34 staining. **(B)** c-kit staining. Bar indicates 100 μm.

**Figure 3:**
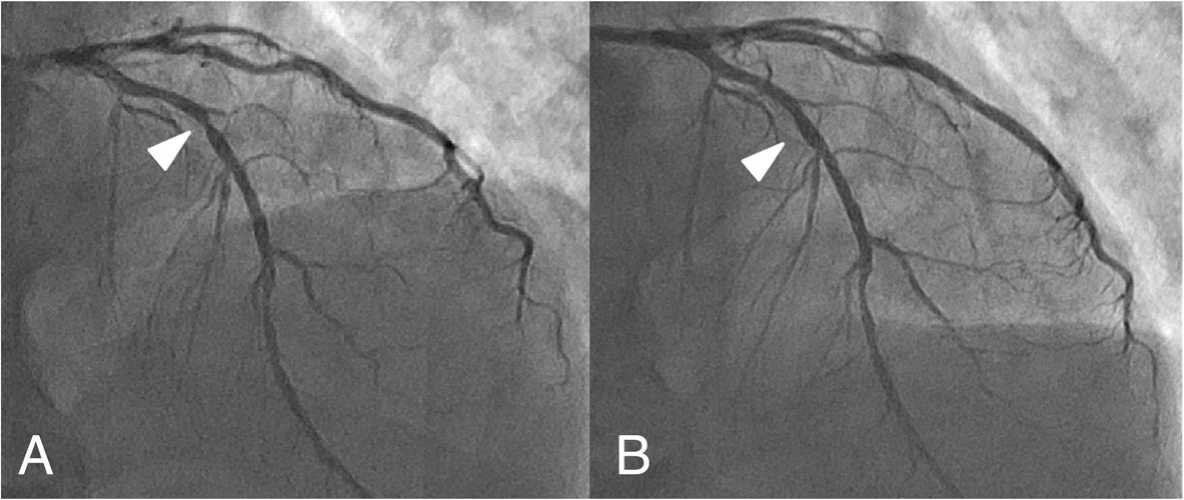
**Straight cranial view of coronary arteriogram (CAG). (A)** Before stenting, the proximal portion of the left anterior descending coronary artery showed 90% stenosis as indicated by the arrowhead. **(B)** After the coronary intervention, the stent was patent.

A 74-year-old man visited our department complaining of melena and an anal mass. On digital examination, a firm, round-shaped, rubbery-textured mass was palpated at the anterior wall of the rectum 2 cm from the anal verge. The mass was adhesive to the prostate, but not to the pelvic floor. Computed tomography (CT) scans and magnetic resonance imaging (MRI) of the pelvis showed a mass 45 mm in diameter between the anterior wall of the lower rectum and prostate (Figure 1A,B and image not shown, respectively). Colonoscopy showed a submucosal mass with a shallow depression in the center just above the dentate line. Fine needle biopsy revealed bundles of spindle-like cells, with 5 mitotic cells per 50 high-power fields, and a Ki-67 (MIB-1) labeling index of 5 to 8%. The tumor was both CD34-positive and c-kit-positive (Figure 2), which suggested a GIST.He had a medical history of diabetes mellitus and dyslipidemia with severe coronary artery calcification, which was detected by a chest CT scan. He performed a treadmill exercise stress test, the results of which indicated comorbid ischemic heart disease. Coronary angiography (CAG) was also performed, and revealed multiple coronary artery stenoses; 75 to 90% stenosis in the left anterior descending artery (LAD; Figure 3A) and 90% stenosis in the left circumflex artery. Percutaneous coronary intervention (PCI) was performed using a drug-eluting stent (Endeavor; Medtronic Cardiovascular Inc., Santa Rosa, CA, USA) for the LAD lesion.The patient subsequently administered imatinib (400 mg daily), aspirin (100 mg daily), and clopidogrel (75 mg daily) orally. Follow-up CAG showed excellent patency at the stenting site and no obvious change in other segments 6 months after the coronary intervention (Figure 3B). Meanwhile, follow-up image studies showed that the tumor was gradually decreasing in size. A final evaluation revealed a 24 mm mass between the rectum and prostate (Figure 1C,D), which appeared to provide an adequate surgical margin for anal sphincter-sparing surgery after 12 months of imatinib treatment. The patient did not exhibit either imatinib- or anti-coagulant-related adverse events.

Laparoscopic low anterior resection with diverting ileostomy was performed after a week preoperative imatinib-free interval. The patient exhibited urinary retention and ileus shortly after surgery, both of which were ameliorated conservatively, and no postoperative cardiac event was noted. Histopathological examinations revealed that CD34-positive spindle-shaped eosinophilic tumor cells spread from the muscularis propria to the adventitia with marked hyalinization (image not shown). The tumor was diagnosed as a GIST of low-grade malignancy due to the negligible number of mitotic cells per 50 high-power fields. Surgical margins were proven clear of viable tumor cells. The patient remains free of recurrence 1 year after surgery without adjuvant chemotherapy.

## Conclusions

GISTs of the colon and rectum are relatively uncommon, and only account for approximately 5 to 10% of GISTs in the gastrointestinal tract [1]. Patients with GISTs of the colon and rectum have been shown to have a poorer outcome than those with GISTs in the stomach or small intestine [3].

Surgical resection is the mainstay of treatment for localized GISTs [1,2]. However, complete resection of rectal GISTs can be difficult because they are often large and may adhere to the surrounding organs or pelvic floor. Pretreatment with imatinib has been an attractive option for GISTs if *en bloc* resection is impossible because of the bulkiness or anatomical location of the tumor. Fiore et al. reported that all patients with GISTs showed a median tumor size reduction of 34% following preoperative imatinib therapy for a median of 9 months with tolerable toxicities [4]. A phase 2 trial demonstrated that imatinib treatment for only one week could cause a reduction in the maximal standardized uptake value on 18-fluorodeoxyglucose positron emission tomography or decrease in blood flow in 60 to 70% of gastrointestinal GIST cases [5]. Previous studies reported a response rate of 73 to 78% with no progressive disease following preoperative imatinib therapy for 1 to 60 months [6-8]. Importantly, the clinical response to imatinib depends on the mutational status of c-kit and platelet-derived growth factor receptor alpha (PDGFRA). The response rate was 84% for patients with GISTs containing an exon-11 mutation, whereas it was 48% for those with an exon-9 mutation or no detectable c-kit or PDGFRA mutation. On the other hand, imatinib was not effective for GISTs harboring mutations in exon 13 or 17 [9]. Clinicians should be reminded of these potential non-responders in neoadjuvant imatinib therapy.

Although controversy still remains regarding the optimal duration of neoadjuvant imatinib therapy, the drug has typically been administered for 6 to 12 months [2,10]. This period has been designed based on the finding that the median time to achieve a maximum tumor response was 4 months and most responses occurred within 9 months of therapy in a phase 3 randomized controlled trial of imatinib for unresectable GISTs [11]. Since resistance to imatinib has been reported, close monitoring is crucial to achieve the best surgical timing; otherwise the opportunity for surgical excision may be missed [4].

There are an increasing number of cases of gastrointestinal malignancies with major comorbidities [12]. The presence of chronic illnesses can affect the effectiveness of and tolerance to the treatment, and is also associated with poorer outcomes following colorectal surgery [13,14]. Our patient had coronary heart disease that required a percutaneous coronary intervention. A recent meta-analysis demonstrated that drug-eluting stents were associated with lower repeat revascularization than that of bare metal stents with no increase in mortality, stent thrombosis, or recurrent myocardial infarction in patients undergoing primary percutaneous coronary intervention [15]. Several guidelines recommend dual antiplatelet therapy over a long time period (e.g., 6 months) following drug-eluting stent implantation [16,17]. This duration matched that of neoadjuvant imatinib therapy in this case.

Radical resection (R0) is associated with the postoperative prognosis for GISTs [2]. Therefore, laparoscopic surgery, a minimally invasive treatment option, is beneficial because its magnified view provides a more precise image of the area to be dissected, and consequently improves oncological quality. Only a limited number of cases of GISTs resected by laparoscopic surgery following imatinib therapy have been reported to date [18,19]. Fujimoto et al. described five successful cases of laparoscopic intersphincteric resection for rectal GISTs following imatinib treatment for 4 to 12 months. All patients survived free of relapse in the 13 to 51 months of follow-up [20]. They mentioned that laparoscopic surgery was found to be minimally invasive, safe, and feasible for downsized GISTs, which our case report further exemplified.

In summary, our case newly demonstrated that neoadjuvant imatinib therapy could provide additional time for the treatment of other major comorbid illnesses, in addition to increasing the possibility of R0 dissection and minimizing the risk of rupture during surgical manipulation, which improve patient prognosis as previously proposed [2,7,8].

## Consent

Written informed consent was obtained from the patient for publication of this Case report and any accompanying images. A copy of the written consent is available for review by the Editor-in-Chief of this journal.

## Abbreviations

CT: Computed tomography; CAG: Coronary angiography; DES: Drug-eluting stent; GIST: Gastrointestinal stromal tumor; LAD: Left anterior descending artery; MRI: Magnetic resonance imaging; PCI: Percutaneous coronary intervention; PDGFRA: Platelet-derived growth factor receptor alpha.

## Competing interests

The authors declare that they have no competing interests.

## Authors’ contribution

HN wrote the manuscript. TK, TT, SI, ES, JK, and TW performed the operation. MT performed the percutaneous coronary intervention, participated in the acquisition and interpretation of radiological data, chose figures, and contributed to the final revision. MI carried out the histological studies. MI, IK, and WT reviewed the paper. All authors read and approved the final manuscript.
